# Supplementary material for: The Evolution and Characterization of the RNA Interference Pathways in Lophotrochozoa
Source: Genome Biol Evol. 2024 May 7;16(5):evae098. doi: 10.1093/gbe/evae098 (PMC11114477; doi:10.1093/gbe/evae098)
Supplement: evae098_Supplementary_Data [file evae098_supplementary_data.zip › Supplementary_materials.zip!/selected_miRNAs/DaRe_selected_miRNas/DaRe_NC_007131.7_34563.pdf]

Provisional ID	:	NC_007131.7_34563
Score total	:	14.1
Score for star read(s)	:	3.9
Score for read counts	:	6.8
Score for mfe	:	2.4
Score for randfold	:	1.6
Score for cons. seed	:	-0.6
Total read count	:	25
Mature read count	:	19
Loop read count	:	0
Star read count	:	6

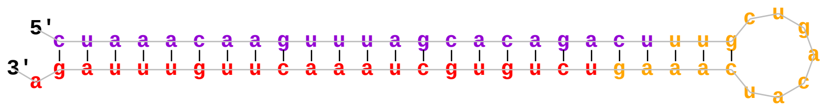

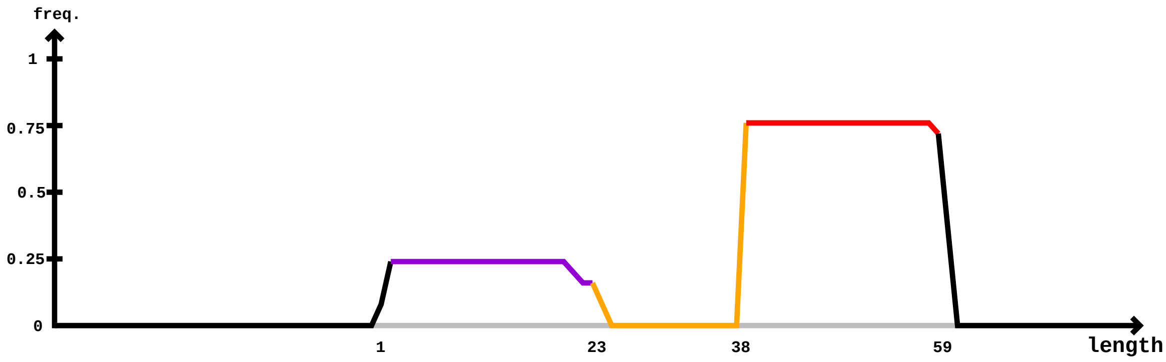

Star Mature

```
5'- cgcaaacugcguacauucuaauaaacagcuauucuaaacaaguuuagcacagacuuugcugacaucaaagucugugcuaaacuuguuuagaauagcuguuuuuuucauauu  -3'  obs
    cgcaaacugcguacauucuaauaaacagcuauucuaaacaaguuuagcacagacuuugcugacaucaaagucugugcuaaacuuguuuagaauagcuguuuuuuucauauu       exp
    ((((...)))).............((((((((((((((((((((((((((((((((((((.......))))))))))))))))))))))))))))))))))))..........  reads  mm  sample
    .................................cuGaacaaguuuagcacagacu........................................................  1      1   Ov2
    ......................................................................ucugugcuGaacuuguuuaga....................  1      1   Ov2

    ......................................................................ucugugcuaaacuuguuuaga....................  1      0   Ov1

    ...............................ucuGaacaaguuuagcacaga...........................................................  1      1   Gu3
    .................................cuGaacaaguuuagcacagacu........................................................  1      1   Gu3
    ......................................................................ucugugcuaaacuuguuuaga....................  3      0   Gu3
    ......................................................................ucugugcuaaacuuguuuGga....................  1      1   Gu3
    ......................................................................ucugugcuaaacuuguuuagaa...................  1      0   Gu3

    ...............................ucuGaacaaguuuagcacag............................................................  1      1   Gu1
    .................................cuGaacaaguuuagcacagacu........................................................  1      1   Gu1
    ......................................................................ucugugcuaaacuuguuuag.....................  1      0   Gu1
    ......................................................................ucugugcuaaacuuguuuaga....................  6      0   Gu1
    ......................................................................ucugugcuGaacuuguuuaga....................  1      1   Gu1

    ......................................................................ucugugcuGaacuuguuuaga....................  1      1   Ov3
    ......................................................................ucugugcuaaacuuguuuagaa...................  1      0   Ov3

    .................................cuGaacaaguuuagcacagacu........................................................  1      1   Gu2
    ......................................................................ucugugcuaaacuuguuuaga....................  2      0   Gu2
```
